# Protection against Myocardial Ischemia-Reperfusion Injury at Onset of Type 2 Diabetes in Zucker Diabetic Fatty Rats Is Associated with Altered Glucose Oxidation

**DOI:** 10.1371/journal.pone.0064093

**Published:** 2013-05-21

**Authors:** Jonas Agerlund Povlsen, Bo Løfgren, Christian Dalgas, Rune Isak Dupont Birkler, Mogens Johannsen, Nicolaj Brejnholt Støttrup, Hans Erik Bøtker

**Affiliations:** 1 Department of Cardiology, Aarhus University Hospital, Skejby, Aarhus N, Denmark; 2 Institute of Clinical Medicine, Aarhus University, Aarhus, Denmark; 3 Department of Forensic Medicine, Aarhus University, Aarhus, Denmark; Pennington Biomedical Research Center, United States of America

## Abstract

**Background:**

Inhibition of glucose oxidation during initial reperfusion confers protection against ischemia-reperfusion (IR) injury in the heart. Mitochondrial metabolism is altered with progression of type 2 diabetes (T2DM). We hypothesized that the metabolic alterations present at onset of T2DM induce cardioprotection by metabolic shutdown during IR, and that chronic alterations seen in late T2DM cause increased IR injury.

**Methods:**

Isolated perfused hearts from 6 (prediabetic), 12 (onset of T2DM) and 24 (late T2DM) weeks old male Zucker diabetic fatty rats (ZDF) and their age-matched heterozygote controls were subjected to 40 min ischemia/120 min reperfusion. IR injury was assessed by TTC-staining. Myocardial glucose metabolism was evaluated by glucose tracer kinetics (glucose uptake-, glycolysis- and glucose oxidation rates), myocardial microdialysis (metabolomics) and tissue glycogen measurements.

**Results:**

T2DM altered the development in sensitivity towards IR injury compared to controls. At late diabetes ZDF hearts suffered increased damage, while injury was decreased at onset of T2DM. Coincident with cardioprotection, oxidation of exogenous glucose was decreased during the initial and normalized after 5 minutes of reperfusion. Metabolomic analysis of citric acid cycle intermediates demonstrated that cardioprotection was associated with a reversible shutdown of mitochondrial glucose metabolism during ischemia and early reperfusion at onset of but not at late type 2 diabetes.

**Conclusions:**

The metabolic alterations of type 2 diabetes are associated with protection against IR injury at onset but detrimental effects in late diabetes mellitus consistent with progressive dysfunction of glucose oxidation. These findings may explain the variable efficacy of cardioprotective interventions in individuals with type 2 diabetes.

## Introduction

Patients with type 2 diabetes seem to have increased sensitivity toward ischemia-reperfusion (IR) injury and attenuated ability to activate endogenous cardioprotection against IR injury [1], but the results from clinical [2] and experimental studies [3] are not consistent.

Mitochondria are the end-effectors of various cardioprotective strategies. Downregulation of metabolism facilitates cardioprotection [4]. Metabolic shut-down and gradual wake-up by extending the endogenous inhibition of mitochondrial respiration from ischemia to initial reperfusion followed by gradual reversal during subsequent reperfusion mediates cardioprotection [5], because the burst of reactive oxygen species (ROS) and Ca^2+^ overload prompted by unmodified postischemic reperfusion are attenuated [6]. Reduced glycolysis rate and inhibition of respiratory complexes during ischemia are inherent parts of ischemic preconditioning (IPC) [7]. Mitochondrial dysfunction is considered inherent to the pathophysiology of type 2 diabetes [8] and may diminish the metabolic flexibility, which is a prerequisite for modification of metabolism to elicit cardioprotection.

We have recently shown that inhibition of mitochondrial metabolism during ischemia and early reperfusion, by blockage of the malate-aspartate shuttle (MAS), elicits a cardioprotective effect similar to IPC [9]–[11]. MAS constitutes an important mechanism for transport of reduced equivalents from the cytosol to the mitochondria for oxidation and facilitation of glucose oxidation. Its activity is regulated by substrate availability and specific aspartate/glutamate carrier (AGC) proteins located in the mitochondrial membrane [12]. AGC contributes to an asymmetric distribution of reduced nicotine adenine dinucleotide between the cytosolic and the mitochondrial compartment and consists of two proteins, citrin and aralar, of which aralar is predominant [13]. Overexpression of aralar has been shown to increase glucose oxidation and mitochondrial activity in pancreatic beta cells [14]. In addition, glutamate transfer across the mitochondrial membrane by the excitatory amino acid transporter 1 (EAAT1) is thought to involve MAS. EAAT1 may account for an increase in MAS activity in hyperthyroid rats [15], suggesting that it may adapt to various pathological conditions. We have recently demonstrated decreased expression of EAAT1 in Zucker Diabetic Fatty (ZDF) rat hearts [16]. The influence of diabetes duration on the myocardial glucose metabolism during IR and the integrated MAS expression influencing key metabolic pathways for cardioprotection is unknown.

The aim of the present study was to investigate whether sensitivity toward IR injury in type 2 diabetes was dependent on the duration of diabetes, and if so whether differences in sensitivity were associated with changes in myocardial glucose metabolism during ischemia and reperfusion.

## Materials and Methods

### Ethics Statement

Animals were handled in accordance with national and institutional guidelines for animal research. The experimental work was approved by the Danish Animal Experiments Inspectorate (license no. 2011/561-2010-C2).

### Animals

Male Zucker diabetic fatty (ZDF) rats (homozygote (fa/fa)) and their age-matched lean controls (fa/+) (Charles River Laboratories, Kisslegg, Germany) were studied at ages 6, 12 and 24 weeks corresponding to a prediabetic state, onset of and late type 2 diabetes. The rats did not receive any anti-diabetic treatment. They were fed Purina 5008 diet as recommended by the supplier and housed under controlled conditions with 12∶12 h light-dark cycles.

Animals were subjected to 12–16 hours of fasting before experimental procedures. All rats were anaesthetized with Dormicum® (midazolam, 0.5 mg/kg body weight (bwt), Matrix Pharmaceuticals, Herlev, DK) and Hypnorm® (fentanylcitrate, 0.158 mg/kg bwt and fluanisone, 0.5 mg/kg bwt, Vetapharma Ltd., Leeds, UK) by subcutaneous injection before excision of the heart for Langendorff perfusion or tissue analyses.

### Study Design and Experimental Protocol

**Figure 1:**
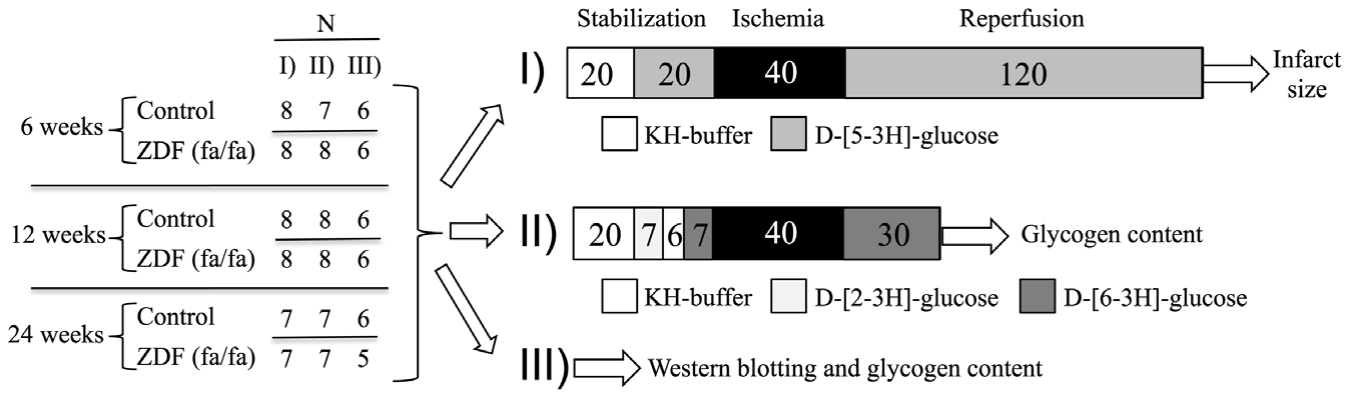
Study design and experimental protocol. Overview of groups, number of animals (**N**) and experimental protocols. **KH-buffer:** Krebs-Henseleit buffer.

The study consisted of three arms that encompassed the following analyses (Figure 1):

**I. IR-sensitivity analyses**; IR with determination of infarct size (IS), hemodynamic performance, myocardial microdialysis and exogenous glycolysis rate.

**II. Tracer analyses+postischemic tissue analyses**; IR with determination of glucose uptake rate during stabilization and glucose oxidation rate during stabilization and initial reperfusion+myocardial content of total-glycogen after 30 min reperfusion.

**III. Baseline tissue analyses**; myocardial protein expression of MAS-related proteins+measurement of baseline total-glycogen.

### Analysis of Blood Glucose and Plasma Metabolites

Preanesthetic fasting blood samples were drawn on animals included in the IR sensitivity analyses (**I**) for measurements of fasting blood glucose (OneTouch® Ultra Blood Glucose, Lifescan Inc., CA, USA) and plasma insulin (DRG Instruments, Marburg, Germany). Samples were obtained from tail-vein bleeding. Preparation for insulin analyses included collection in heparinized capillary tubes (∼100 µL), centrifugation (5000 RPM for 1 min at ambient temperature), collection of the supernatant and storage at −80°C before analysis with a hypersensitive rat insulin ELISA kit (DRG Instruments, Marburg, Germany). Serum total-cholesterol, triglyceride and free fatty acids were measured on blood samples drawn from the aorta of the animal immediately before excision of the heart. Preparation included centrifugation (3500 RPM for 10 min at 4°C), collection of the supernatant and storage at −80°C until analysis on a Cobas Integra Analyzer (Roche Diagnostics, Rotkreuz, Switzerland).

### Isolated Perfused Rat Heart Model

Hearts **(I+II)** were cannulated *in-situ* without disruption of coronary flow and perfused retrogradely a.m. Langendorff with Krebs-Henseleit (KH) solution (11 mM glucose) at a constant pressure of 80 mmHg, as described in detail previously [17]. The perfusion protocol consisted of 40 min stabilization, 40 min global ischemia and 120 min reperfusion. Left ventricular function was measured by an intraventricular balloon connected to a pressure transducer and coronary flow (CF) by an inline flowmeter (Hugo Sachs Electronic, March-Hugstetten, DE). Diastolic pressure was set to 5–8 mmHg. Data were acquired and analyzed with Notochord®-hem software (Notocord, Croissy-Sur-Seine, France). Hearts were frozen, sliced and underwent vital staining with 2,3,5-triphenyltetrazoliumchloride to delineate areas of infarction **(I)**. IS was assessed by manual delineation using image analysis software (ImageJ, NIH). Measurements were weighted with the weight of each individual slice. The IS/area-at-risk (IS/AAR) ratio was then calculated for each heart.

### Myocardial Microdialysis

Myocardial microdialysis was performed on all hearts in the perfusion analyses (**I**) to assess interstitial concentrations of citric acid cycle intermediates and glycolytic end products, as described previously [9]. In brief, a microdialysis probe (membrane length 4 mm, cut-off 6 Da; AgnTho’s AB, Sweden) was inserted into the free left ventricular wall of the myocardium enabling sampling with a perfusion rate of 1 µL/min over 10 min. Perfusion fluid was KH buffer deoxygenated with 95% N_2_ and 5% CO_2_. Microdialysis samples were separated by ultra-performance liquid chromatography using a Waters Xevo TQ-S triple quadrupole tandem mass spectrometer (Waters Corp., Manchester, UK). Analytes were verified using two daughter ions and internal standards when possible [18]. Results were corrected for relative recovery rates that have been determined previously [9], [18].

### Exogenous Glucose Metabolism

Rates of glucose uptake, glycolysis and glucose oxidation were measured using statically labeled tritiated glucose isotopes [19]. A buffer volume of 1500 mL (5 mCi/100 mL) was recirculated. Preexperimental buffer samples were drawn to assess baseline specific activity (SA) pr. µmol glucose in the buffer. Glucose uptake was assessed by ^3^H_2_O production from D-[2-3H]-glucose [20], [21]. Glycolysis rate was determined by liberated ^3^H_2_O from D-[5-3H]-glucose at the enolase step of glycolysis [22]. Glucose oxidation was quantified by ^3^H_2_O production by oxidation of D-[6-3H]-glucose in the citric acid cycle [19]. Because the buffer was recirculated, samples from the inline flow tube (“arterial” (A)) and the coronary effluent (“venous” (V)) were collected and analyzed to assess ^3^H_2_O production. This was done by separation of labeled glucose from ^3^H_2_O by anion exchange chromatography on AG 1-X8 resin columns (Bio-Rad, Hercules, CA, USA) according to the manufacturer’s instructions. The purified ^3^H_2_O was suspended in 10 mL Opti-Phase scintillation solution (Perkin-Elmer, Shelton, CT, USA) and quantified by beta-scintillation on a TriCarb 2900TR liquid scintillation analyzer (Packard, Perkin, IL, USA) in detections pr. minute (dpm). Rates of glucose utilization corrected for heart weight and sampling time [19] were calculated as follows:
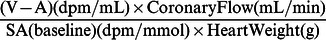



### Myocardial Total-glycogen

Analyses were done on snap-frozen biopsies removed with scissors from the left ventricle in vivo (**III**) and after 30 minutes of reperfusion (**II**). Total glycogen was determined in triplicates after degradation to glucose using the filter-paper technique and spectrophotometric detection [23].

### Western Blotting

Left ventricular biopsies were homogenized in 2 mL lysis-buffer [16] and subsequently centrifuged for 15 min at 4°C and 1000 G. Gel samples were prepared from the supernatant and total protein concentration determined with a Pierce BCA Protein Assay Reagent (Thermo Fisher Scientific Inc., Rockford, USA).

Samples were run on 12.5% polyacrylamide gels (Criterion Tris-HCl, Bio-Rad, CA, USA) and subsequently transferred to a nitrocellulose membrane. The blots were blocked with milk, washed in PBS-T and incubated with primary antibodies overnight at 4°C. Following incubation with horseradish peroxidase-conjugated secondary antibody (P448, diluted 1∶3000, DAKO, Glostrup, Denmark) antigen-antibody complexes were visualized with enhanced chemiluminiscense system (Amersham Pharmacia Biotech, Denmark). Densitometry of protein bands was performed, and for each gel an identical gel was run and subjected to Coomassie staining to ascertain identical loading or to allow for potential correction for minor differences in loading after scanning and densitometry.

#### Antibodies

The following primary antibodies (rabbit polyclonal) were used: Aralar - (1∶2000) (ab90095, Abcam, Cambridge, UK); Citrin – (1∶500) (sc-98624, Santa Cruz, CA, USA); EAAT1– (1∶1000) (ab416, Abcam, Cambridge, UK); GOT1/GOT2– (1∶1000/1∶2000) (ARP48205_T100/ARP43517_T100); MDH1/MDH2– (1∶1500/1∶1000) (ARP48284_T100/ARP48286_T100, Aviva Systems Biology, CA, USA) When available, a blocking peptide was used as a negative control.

### Statistics

For reasons of clarity all data are reported as mean ± SEM, unless stated otherwise, even when the differences between groups were tested with a non-parametric test. Data were analyzed to assess age effect within control and ZDF groups and the effect of diabetes by comparing ZDF groups with age-matched control groups by two-way ANOVA followed by a pairwise comparison by post hoc Bonferroni modification when appropriate. Non-parametric data were compared with Kruskal-Wallis test. Data containing repeated measurements were analyzed using repeated measurements ANOVA. Calculations were performed using GraphPad Prism (GraphPad Software, CA, USA). P<0.05 was considered statistically significant.

## Results

### Animal Characteristics

Heart-, body weights and biochemical characteristics are summarized in Table 1. ZDF rats had increased body weight compared to their respective controls at 6 and 12 weeks of age, while body weight was decreased at 24 weeks of age. Heart weight was similar in control and ZDF rats at 6 and 12 weeks and significantly lower at 24 weeks. In both control and ZDF rats heart weight increased with age. Heart weight corrected for body weight was smaller in ZDF rats at all ages.

**Table 1:** Animal characteristics.

	6 weeks		12 weeks		24 weeks	
	Control (n = 8)	ZDF (n = 8)	Control (n = 8)	ZDF (n = 8)	Control (n = 7)	ZDF (n = 7)
Body weight (BW), g	179±3	204±6*	317±3†	362±7* †	446±4†	423±11* †
Heart weight (HW), mg	748±26	730±17	1100±41†	1101±29†	1509±64†	1258±32* †
HW/BW, ratio	4.17±0.11	3.59±0.08*	3.48±0.13†	3.05±0.07* †	3.38±0.13†	2.98±0.09* †
B-glucose, mmol/L	4.4±0.1	5.3±0.2*	4.7±0.1	13.1±1.6* †	5.4±0.1†	23.4±1.5* †
P-insulin, pmol/L	6.4±1.0	331.3±40.7*	21.8±5.1	298.0±27.9*	66.1±10.4	92.1±20.1†
S-total cholesterol, mmol/L	1.67±0.09	1.89±0.17	1.59±0.06	3.38±0.13* †	2.47±0.33	7.47±0.60* †
S-triglyceride, mmol/L	0.49±0.03	2.42±0.25*	0.51±0.03	6.57±0.60* †	0.74±0.06	10.68±1.32* †
S-free fatty acid, mmol/L	1.40±0.14	4.55±0.80*	1.37±0.12	5.54±0.80*	2.36±0.23	7.75±1.76* †

Mean±SEM.

* p<0.05 compared to age-matched controls.

† p<0.05 compared to 6 weeks control/ZDF.

Albeit still within the normoglycemic range, preoperative fasting glucose was elevated at 6 weeks in ZDF compared to control rats. At 12 weeks ZDF rats were hyperglycemic with a further increase at 24 weeks. In ZDF rats there was a ∼50 fold increase in insulin at 6 weeks compared to controls and a subsequent decline at 12 and 24 weeks. Total cholesterol, triglyceride and free fatty acids were elevated at 6 weeks, although non-significant for cholesterol, with a further increase at 12 and 24 weeks in ZDF rats. All parameters increased with age in control rats.

### Preischemic Cardiac Function

**Figure 2:**
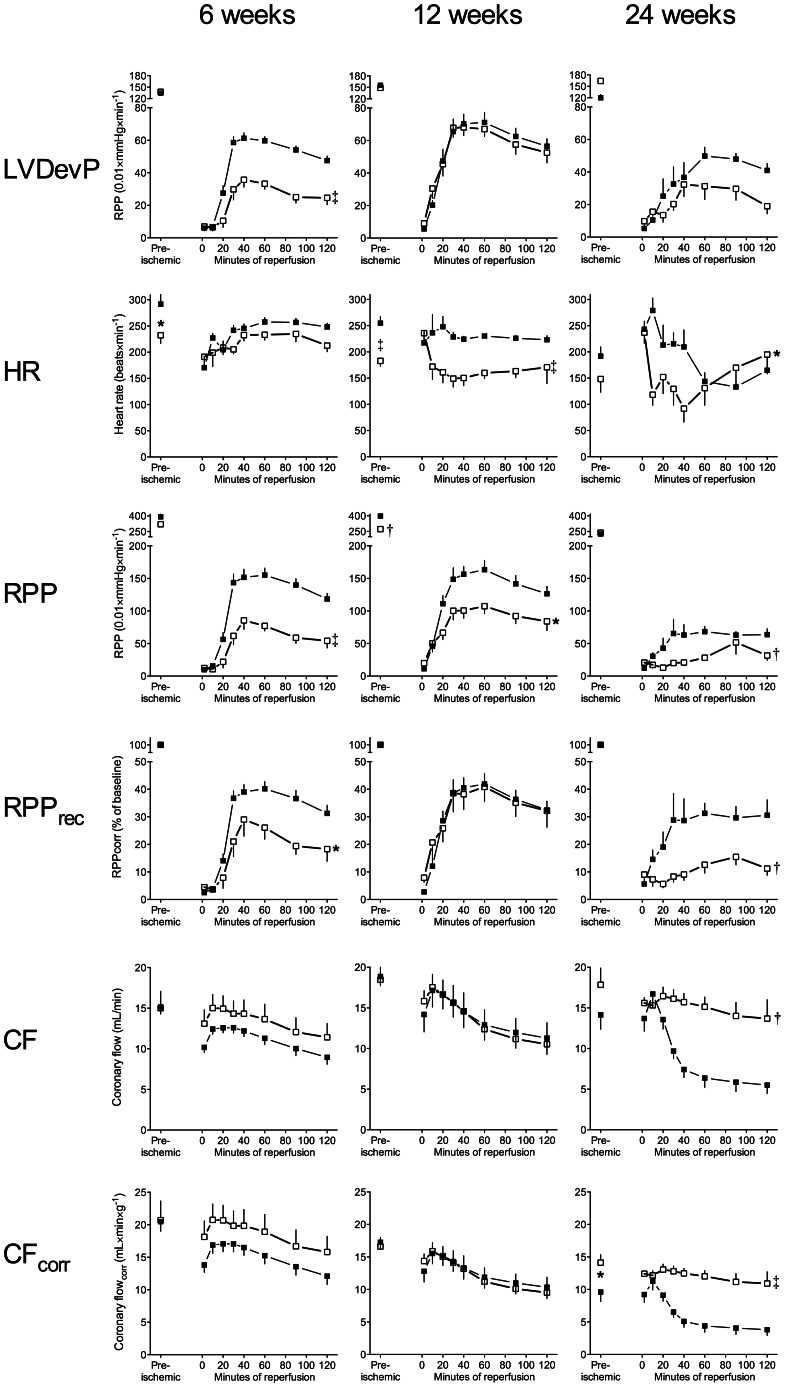
Hemodynamic parameters at baseline and during reperfusion. Hemodynamic performance during stabilization and reperfusion. Mean±SEM. Closed symbols: Control, Open symbols: ZDF. * p<0.05, † p<0.01, ‡ p<0.001. **LVDevP**: Left ventricular developed pressure. **HR**: Heart rate. **RPP**: rate-pressure-product. **RPPrec**: % recovery from baseline rate-pressure-product. **CF_corr_**: Coronary flow corrected for heart weight.

Figure 2 shows preischemic cardiac function. At 6 and 12 weeks control and ZDF hearts had similar left ventricular developed pressure (LVDevP) and CF. ZDF hearts displayed lower intrinsic heart rates (HR) and rate-pressure-product (RPP = HR × LVDevP), although not statistically significant at 6 weeks. At 24 weeks of age there was no difference in RPP, as LVDevP and HR declined in the control group, while LVDevP increased in the ZDF group. The latter was accompanied by a significant increase in CF corrected for HW (CF_corr_).

### Functional Recovery

Changes in LVDevP, RPP, relative recovery of RPP (RPP_rec_; % of baseline), HR, CF and CF_corr_ are shown in Figure 2.

ZDF hearts had decreased RPP at all ages compared to controls. At 6, but not 12, and 24 weeks LVDevP was decreased, whereas HR was decreased at 12 and 24 weeks. When correcting for baseline RPP there was no difference in RPP_rec_ at 12 weeks in contrast to 6 and 24 weeks. There were no differences in CF and CF_corr_ at 6 and 12 weeks, while both were elevated in ZDF hearts at 24 weeks.

RPP decreased in control hearts from 6 and 12 weeks to 24 weeks (p = 0.0003). In ZDF rats a similar pattern was found between 6 and 24 weeks (p = 0.03), while ZDF hearts recovered better at 12 compared to 6 weeks (p = 0.02). There was a significant decline in CF_corr_ from 6 and 12 weeks to 24 weeks in both control and ZDF hearts.

### Infarct Size and Correlation with Blood Glucose

**Figure 3:**
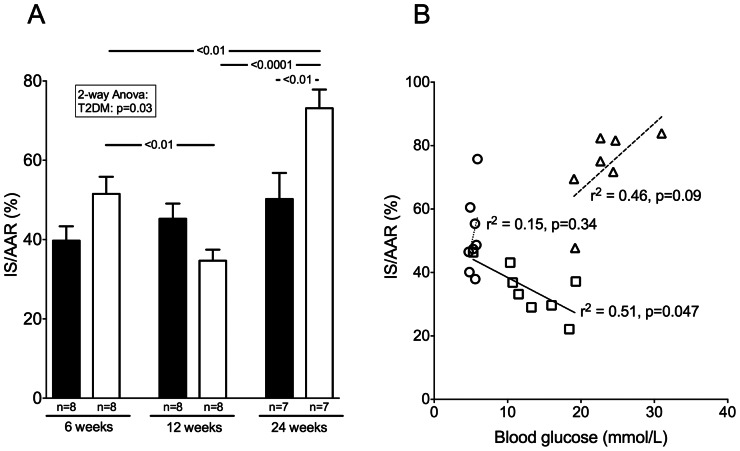
Infarct size and correlation with blood glucose. **A.** Infarct size/area-at-risk ratio (IS/AAR). Black bars: Control, White bars: ZDF. **B.** Correlations between preoperative fasting blood glucose and IS/AAR at prediabetes, onset and late type 2 diabetes mellitus. Mean ± SEM. Circles: ZDF 6 weeks, Squares: ZDF 12 weeks, Triangles: ZDF 24 weeks.

T2DM significantly altered the development in sensitivity towards IR injury compared to controls. At late diabetes ZDF hearts suffered increased IR injury. At onset of T2DM the injury was decreased (Figure 3A).

There was a non-significant increase in IS with increasing age in control hearts. In contrast ZDF hearts had reduced IS at 12 weeks compared to 6 as well as 24 weeks, and IS was significantly higher at 24 than at 6 weeks.

IS correlated inversely with blood glucose concentration at 12 weeks (*r^2^* = 0.51, p = 0.046) and tended to correlate positively at 24 weeks in ZDF rats (*r^2^* = 0.46, p = 0.095) (Figure 3B). There were no significant correlations between IS and circulating free fatty acids, triglyceride or cholesterol concentrations.

### Exogenous Glucose Metabolism

#### Preischemically

**Figure 4:**
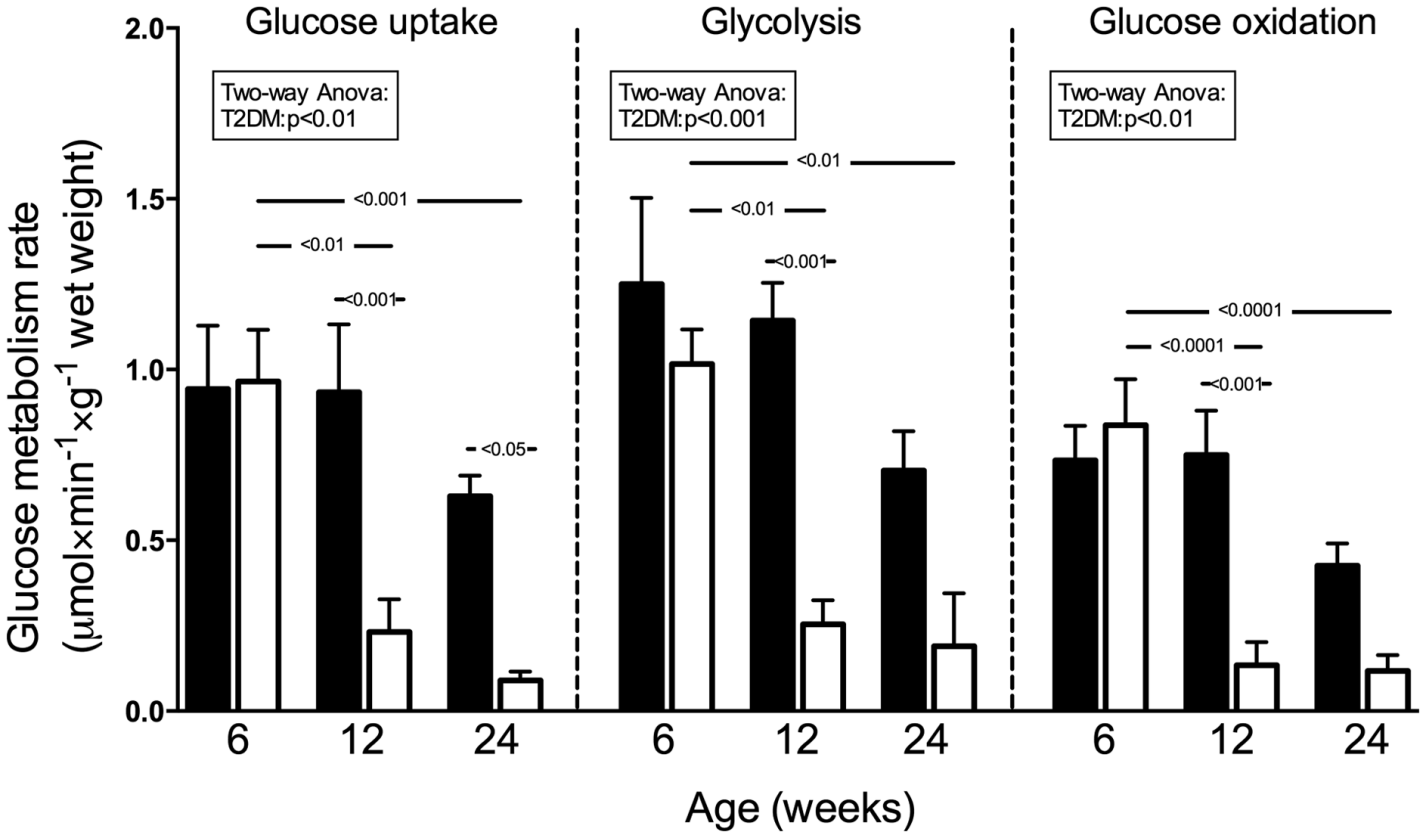
Glucose metabolism prior to ischemia-reperfusion. Glucose uptake – glycolysis- and glucose oxidation rates during stabilization. Mean ± SEM. Black bars: Control, White bars: ZDF.

Rates of exogenous glucose uptake, glycolysis rate and glucose oxidation did not differ between control and ZDF hearts at 6 weeks (Figure 4). At 12 and 24 weeks glucose uptake -, glycolysis- and oxidation rates were reduced in ZDF hearts. All rates decreased with increasing age in ZDF hearts.

#### Postichemically

**Figure 5:**
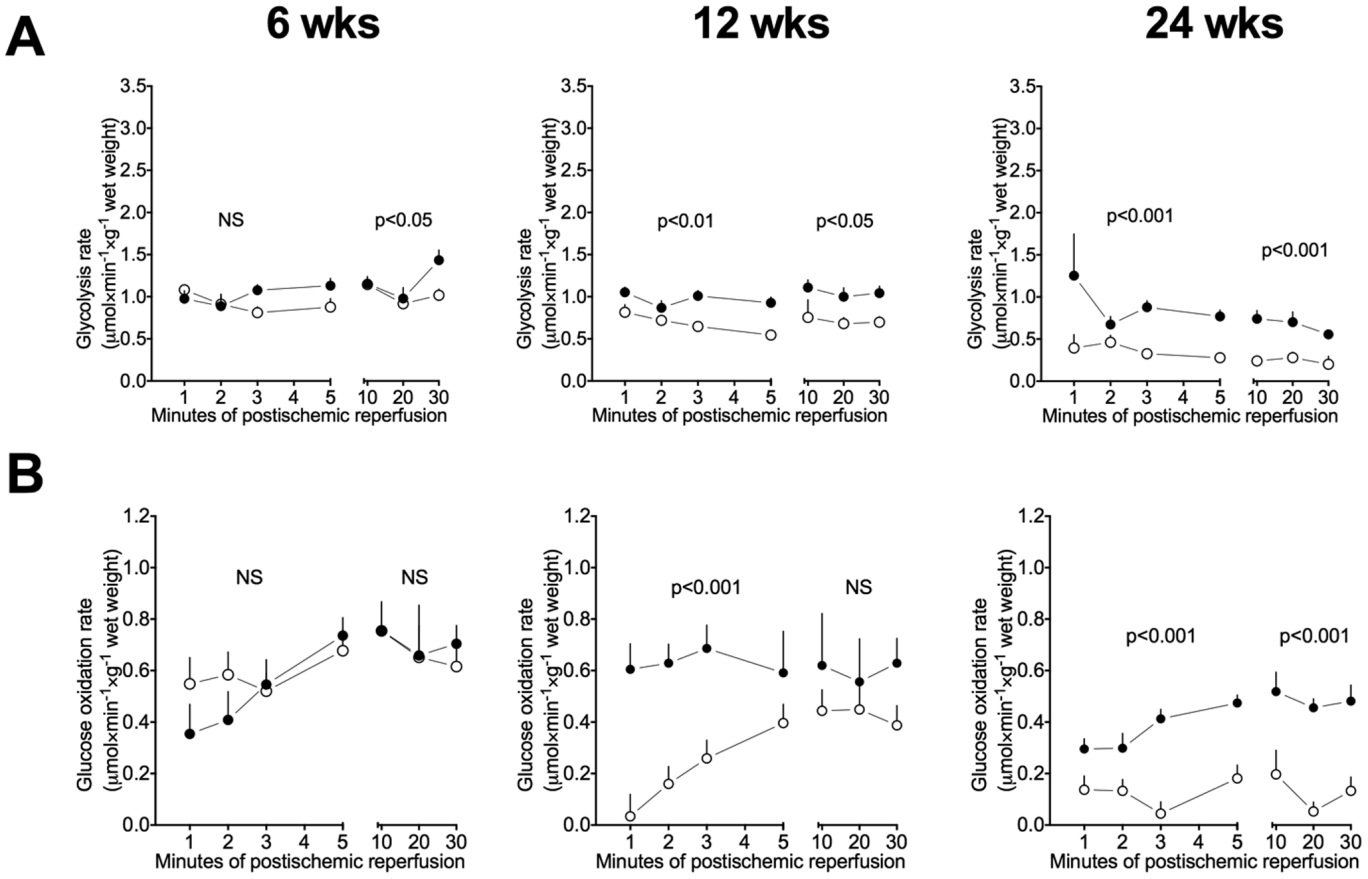
Glucose metabolism during reperfusion. **A**: Glycolysis and **B:** Glucose oxidation rates during reperfusion at 6, 12 and 24 weeks. Mean ± SEM. Closed symbols: Control, Open symbols: ZDF. P-values indicate differences between control and ZDF from 0–5 and 10–30 minutes of reperfusion, respectively.

Compared to control hearts glycolysis rate did not differ at 6 weeks, but decreased at 12 and 24 weeks of age (Figure 5A). Glucose oxidation rates were similar at 6 weeks. At onset of type 2 diabetes (12 weeks) ZDF rats completely shut down their exogenous glucose oxidation during the first few minutes of reperfusion (p = 0.0007) with a subsequent increase to reach control levels between 10–30 minutes of reperfusion (p = 0.12) (Figure 5B). Glucose oxidation was continuously decreased throughout reperfusion in ZDF rats at late diabetes.

### Myocardial Total-glycogen in vivo and after Ischemia-reperfusion

**Figure 6:**
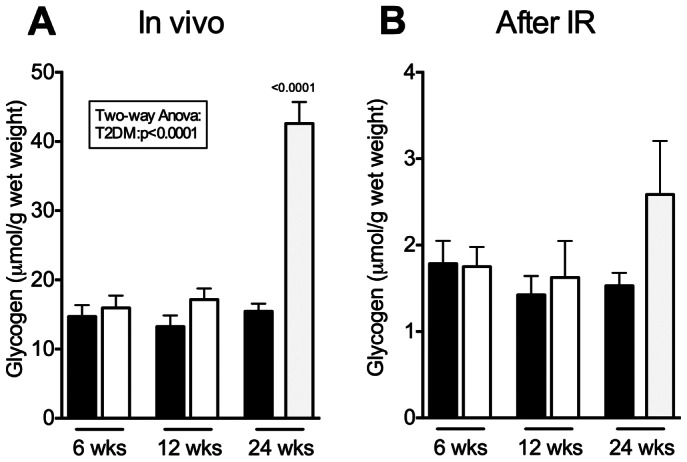
Myocardial glycogen concentrations. Myocardial concentrations of total-glycogen in vivo (**A**) and after ischemia and 30 min reperfusion (**B**) in control and ZDF rats. Mean ± SEM. Black bars: Control, White bars: ZDF. **IR**: ischemia-reperfusion. p<0.0001 vs. age-matched control and ZDF 6 weeks.

In vivo total-glycogen concentrations were similar at 6 and 12 weeks and almost 3-fold increased in ZDF rats at 24 weeks compared to control rats (Figure 6A). After 30 min of reperfusion there were no differences between control and ZDF at any age (Figure 6B).

### Interstitial Metabolite Concentrations during IR

**Figure 7:**
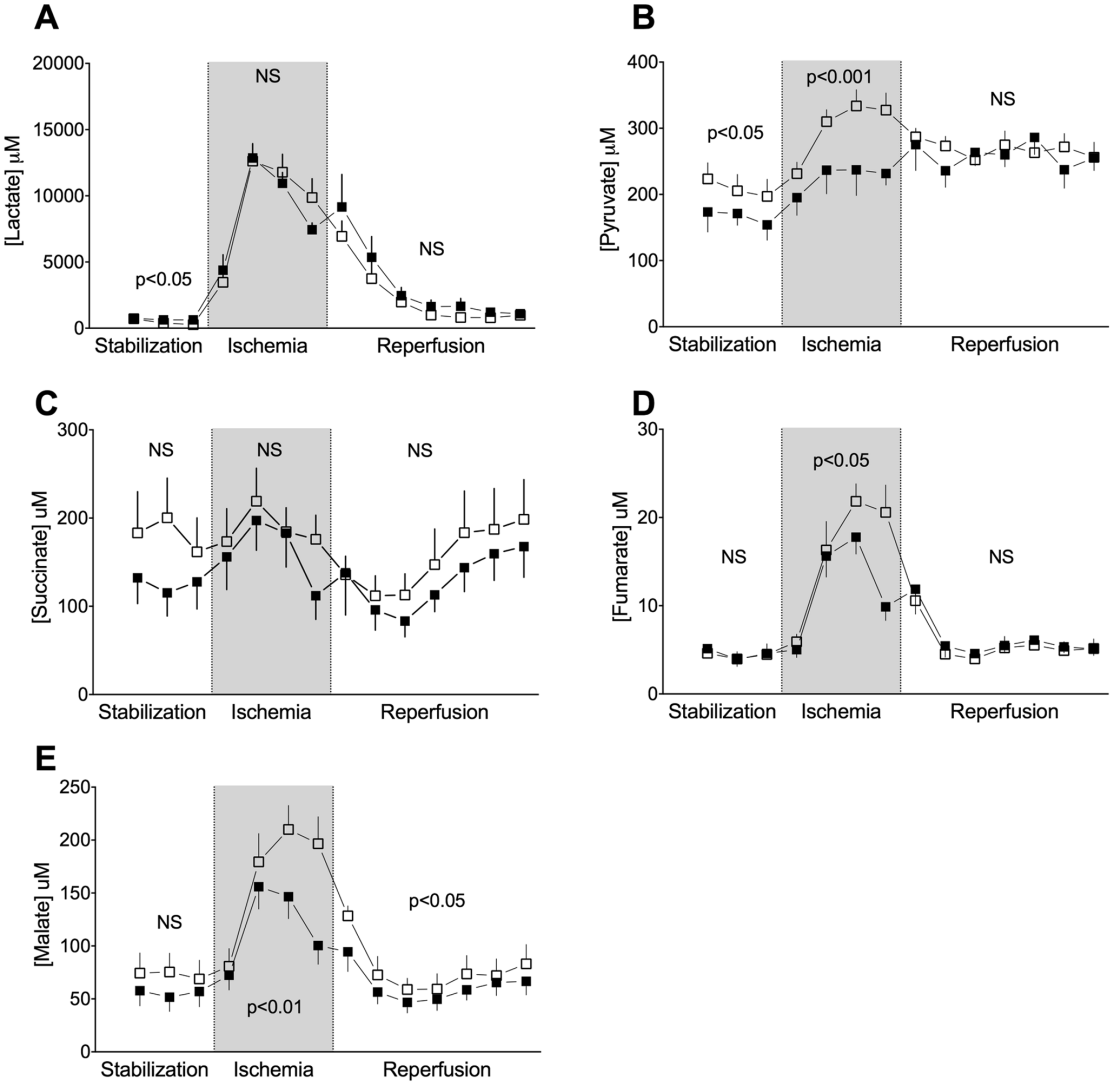
Interstitial concentrations of metabolites. Interstitial concentrations of glycolytic - and citric acid cycle metabolites measured by myocardial microdialysis during ischemia-reperfusion in control (closed squares) and ZDF (open squares) at 12 weeks. Mean ± SEM. **A:** Lactate, **B:** Pyruvate, **C:** Succinate, **D:** Fumarate, **E:** Malate.

Interstitial lactate and pyruvate levels were measurable in all groups during the entire protocol. Fumarate, succinate and malate levels were highest at 12 weeks and quantifiable during the entire protocol in both controls and ZDF rats. At 6 and 24 weeks, concentrations only reached quantitative levels during the last 20 minutes of ischemia and the first 10 minutes of reperfusion. Results for control and ZDF hearts at 12 weeks are shown in Figure 7.

Interstitial lactate levels were similar in control and ZDF hearts during the entire protocol at 6 weeks. ZDF rats had lower interstitial lactate levels during stabilization and the concentration also tended to be lower during reperfusion at 12 weeks (Figure 7A). Interstitial lactate was increased during late ischemia and entire reperfusion in ZDF hearts at 24 weeks (p = 0.02).

ZDF rats had increased concentrations of pyruvate, fumarate and malate during ischemia, and in particular at the end of ischemia, compared to controls at 12 weeks, while succinate was non-significantly increased (Figure 7B–E). During reperfusion malate concentration was increased in ZDF rats, while pyruvate, succinate and fumarate levels were similar. At 6 and 24 weeks there were no differences in pyruvate, fumarate, succinate and malate levels.

### Protein Expression of MAS Transporters and Enzymes

**Figure 8:**
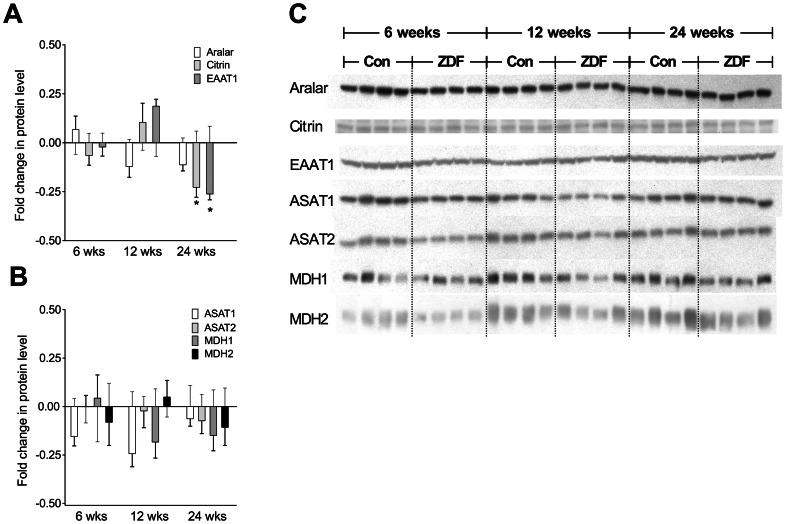
Expression of proteins involved in the malate-aspartate shuttle. Fold change in expression of proteins participating in the malate-aspartate shuttle. Controls are set to 0 at all ages. Bars indicate fold change in ZDF hearts compared to control hearts. **A:** Transporter proteins. **B:** Enzymes. C: Representative western blots. There were no significant changes in expression at 6 and 12 weeks, while citrin and EAAT1 were significantly, and aralar non-significantly, downregulated at 24 weeks of age. Mean ± SEM. **EAAT**: Excitatory Amino Acid Transporter. **ASAT:** Aspartate Amino Acid Transaminase. **MDH:** Malate Dehydrogenase. *p<0.05.

There were no significant alterations in protein expression at 6 and 12 weeks of age, although there was a tendency towards upregulation of EAAT1 and downregulation of aralar and the cytosolic enzymes at 12 weeks. At 24 weeks all transport proteins were downregulated. However, the downregulation of aralar did not reach statistical significance (Figure 8).

## Discussion

This study provides two major findings: 1) sensitivity towards ischemia-reperfusion injury in the type 2 diabetic heart is dependent on the duration of the disease with decreased sensitivity at onset and increased sensitivity at a late stage, 2) this finding was associated with differences in glucose metabolism during ischemia and reperfusion depending on the stage of the disease. At onset of diabetes there was a shutdown of mitochondrial glucose oxidation during ischemia and initial reperfusion with a subsequent gradual awakening. In contrast rats with late type 2 diabetes displayed continuously decreased glucose metabolism throughout reperfusion and downregulated expression of MAS proteins indicating a dysfunctional cytosol to mitochondria communication that should have facilitated gradual awakening of glucose oxidation. Our findings substantiate previous studies on diabetes and IR injury demonstrating the influence of early and late diabetes [24]–[28]. In addition our study examines concomitantly the impact of myocardial glucose metabolism, ranging from glucose uptake to mitochondrial oxidation, at different stages of type 2 diabetes on IR sensitivity.

Heart-, bodyweights and biochemical data confirm that ZDF rats develop characteristics similar to unregulated human type 2 diabetes. The characteristics of our animals are comparable to those reported by Wang et al. [25]. In addition to hyperlipidemia and hypercholesterolemia, ZDF rats develop several obesity- and hyperglycemia-related complications including arterial hypertension, cardiomyopathy, cardiac hypertrophy and nephropathy [29], [30]. Per se, these complications are associated with alterations in either IR sensitivity and/or the effect of cardioprotective strategies. Hyperlipidemia impairs ischemic tolerance and attenuates the effect of conditioning strategies [31], [32]. The effects of compensated hypertrophy and hypertension on IR sensitivity are currently discussed because increased as well as unaltered sensitivity have been reported, while the effect of preconditioning consistently seems to be maintained [33], [34]. In contrast, IR sensitivity is increased in uremia, while the cardioprotective effect of ischemic preconditioning is maintained or even improved [35], [36]. All of these conditions may be involved in the mechanisms behind the findings of our study. However, based on our data it seems that the factors triggering the response to IR injury in diabetic hearts are associated with mitochondrial glucose metabolism possibly through the MAS-cycle. The correlation between myocardial infarct size and preoperative blood glucose concentration indicates that the decrease in sensitivity towards IR injury in early and increase in sensitivity in late diabetes is associated with the severity of hyperglycemia. Although not overtly hyperglycemic, prediabetic rats had higher blood glucose than controls and a borderline increase in IR injury at this stage supporting the impact of preoperative circulating glucose concentration. An influence of hyperglycemia on IR injury in the presence of evident diabetes and a dependency on disease duration gain support from previous studies [24], [37]–[39]. However, these studies have been performed in streptozotocin (STZ) or alloxan induced animal models of type 1 diabetes where the disease, in contrast to type 2 diabetes, develops rapidly without the preceding metabolic abnormalities present in the ZDF rat model. Studies in Goto-Kakizaki (GK) rats, another hereditary animal model of type 2 diabetes, have shown that the dependency of myocardial IR injury on circulating glucose levels is a function of age and associated with concomitant alterations in heart mitochondrial function [40]. Thus, not only the severity but also the duration of hyperglycemia alters sensitivity against IR injury and other yet unidentified mechanisms may be triggered as the animals develop evident diabetes.

The metabolic phenotype associated with reduced sensitivity against IR injury in early diabetes seem to share features with the mechanisms involved in cardioprotection by IPC, which is associated with optimized energy yield by adaptation of major pathways such as glycolysis, glucose - and fatty acid oxidation. Following initial activation of anaerobic glycolysis, persistent ischemia inhibits glycolysis and glycogenolysis. IPC accentuates this inhibition [7], [41] by a metabolic shut-down during ischemia and very early reperfusion followed by a gradual wake-up of metabolism during subsequent reperfusion [5]. Considered a key mechanism behind not only IPC but also cardioprotective strategies like postconditioning and remote ischemic conditioning, the convergence towards mitochondria as the end-effector of ischemic cardioprotection [6], [42] is of importance for type 2 diabetes, because mitochondrial dysfunction is regarded as an inherent part of insulin resistance in type 2 diabetes [8]. Mitochondrial dysfunction compromising metabolic flexibility has also been demonstrated with ageing [43], but the nature of the abnormality and whether it varies with diabetes duration is unknown.

In accordance with previous studies we found elevated tissue concentrations of the citric acid cycle intermediates (CAC) succinate, fumarate and malate during IR injury [44]–[46]. More importantly, we demonstrated increased concentrations during ischemia at onset of type 2 diabetes compared to controls consistent with the cardioprotective effect of fumarate recently demonstrated by Ashrafian et al. [47] in fumarate hydratase (FH) knock-out mice with reduced IR sensitivity. These mice displayed increased levels of second span CAC intermediates (α-ketoglutarate to oxaloacetate) [48], and to compensate for the decreased CAC activity due to FH deficiency they channeled amino acids into the CAC and thereby maintained energetic viability. Our findings are not likely to be caused by a decrease in FH activity, because malate was increased. However, a potential mechanism may be a decreased CAC activity due to decreased consumption of reduced equivalents by the respiratory chain enzymes during early reperfusion. When induced in healthy hearts by respiratory chain enzymes inhibitors, this mechanism induces cardioprotection [4], [49], and a reduced TCA cycle flux has recently been demonstrated in diabetic myotubes [50] supporting this assumption. An alternative or coexistent mechanism could be increased amino acid derived anaplerosis, which would maintain energetic viability [51] and cause an increase in second span metabolites. However, the increased interstitial pyruvate concentration during ischemia in diabetic hearts does not support anaplerosis as a predominant mechanism. The increase in pyruvate concentration rather reflects decreased CAC activity and pyruvate utilization.

Localized in the inner mitochondrial membrane, the MAS constitutes the main pathway for balancing myocardial extra- and intramitochondrial glucose metabolism through translocation of reducing equivalents from the cytosol into the mitochondria. We found decreased expression of ASAT1 and a tendency towards increased expression of the glutamate transporter EAAT1 at onset of diabetes. The association between altered protein expression and dynamic changes in metabolism is unknown. Our findings are consistent with an increased metabolic flexibility at onset of type 2 diabetes allowing initial shut down of glucose oxidation because inhibition of MAS causes attenuated transfer of reduced equivalents and decreased CAC activity during early reperfusion combined with a preserved ability of the shuttle to increase its activity and hence mitochondrial metabolism. An increase in expression of glutamate transporters participating in the MAS has previously been shown to enhance mitochondrial metabolism including glucose oxidation [14]. The increased sensitivity towards IR injury at late diabetes could be a consequence of loss of metabolic flexibility due to exhaustion of the MAS, which showed markedly reduced expression, and severe mitochondrial dysfunction in accordance with a recent human study of cardiac expression of MAS-related proteins on pancreatic islet cells in type 2 diabetes [52]. These abnormalities obstruct the initial metabolic shutdown followed by a gradual acceleration of glucose oxidation [53] and induce irreversible tissue damage [54]. A recently published study by Sárközy et al. [55] confirms that late unregulated diabetes in ZDF rats is associated with extensive changes at the mRNA and protein level of the myocardium. In an exploratory study evaluating ∼15.000 genes in 25 wks old ZDF rats they found profound modifications of the myocardial transcriptome including genes involved in carbohydrate metabolism and membrane transport. Moreover, they demonstrated increased levels of cardiac 3-nitrotyrosine, an indicator of oxidative stress, and regulations of genes related to stress response and oxidative stress, factors that are known to affect IR injury. These and ours findings warrant studies with a mechanistic approach to delineate the pathophysiological consequences of these alterations.

We conducted our experiments in an isolated perfused rat heart model with a circulating glucose concentration of 11 mmol/L and glucose as the sole substrate in the absence of insulin to avoid acute influences of different glucose concentrations and interference from fatty acids (FA) and insulin on IR injury. Sensitivity towards IR injury is substrate dependent with increased injury when metabolizing FA [56], and insulin is an activator of the reperfusion injury salvage kinase pathway, which provides cardioprotection [57]. Wang et al. [25] demonstrated a similar reduction of sensitivity against IR injury in ZDF hearts at onset of type 2 diabetes irrespective of substrate composition. We chose the ZDF rat because it is a commonly used animal model of type 2 diabetes with the inherent metabolic disarrays, i.e. insulin resistance and basal hyperinsulinemia [58], abnormal glucose metabolism [59] and myocardial mitochondrial dysfunction [60], which may modify the efficacy of cardioprotection.

We demonstrated differences in glycolysis and glucose oxidation by quantification of exogenous glucose metabolism. Endogenous glucose metabolism was assessed by tissue measurements of glycogen concentrations before ischemia and after 30 minutes of reperfusion. Although these measurements showed no differences between control and ZDF rats at onset of type 2 diabetes it is a limitation that this method does not allow temporal assessment of endogenous glycogen metabolism during different phases of reperfusion.

A final limitation of our study is that it is not clear to which extend altered protein expression is associated with dynamic changes in metabolism. Although the evidence for the changes in intermediary metabolism and respiratory capacity remain indirect, we applied a variety of approaches and the totality of data from numerous methods underlies our conclusions.

### Conclusions

In conclusion, the alterations in mitochondrial glucose metabolism in type 2 diabetes are associated with protection against IR injury at onset but detrimental effects in late diabetes mellitus. These findings provide an explanation to previous conflicting results on IR injury in individuals with type 2 diabetes.

## References

[1] Ferdinandy P, Schulz R, Baxter GF (2007) Interaction of cardiovascular risk factors with myocardial ischemia/reperfusion injury, preconditioning, and postconditioning. Pharmacol Rev 59: 418–458 doi:10.1124/pr.107.06002. 18048761 10.1124/pr.107.06002

[2] Bøtker HE, Kharbanda R, Schmidt MR, Bøttcher M, Kaltoft AK, et al (2010) Remote ischaemic conditioning before hospital admission, as a complement to angioplasty, and effect on myocardial salvage in patients with acute myocardial infarction: a randomised trial. Lancet 375: 727–734 doi:10.1016/S0140-6736(09)62001-8. 20189026 10.1016/S0140-6736(09)62001-8

[3] Jensen RV, Støttrup NB, Kristiansen SB, Bøtker HE (2012) Release of a humoral circulating cardioprotective factor by remote ischemic preconditioning is dependent on preserved neural pathways in diabetic patients. Basic Res Cardiol 107: 285 doi:10.1007/s00395-012-0285-1. 22821347 10.1007/s00395-012-0285-1

[4] Chen Q, Hoppel CL, Lesnefsky EJ (2006) Blockade of electron transport before cardiac ischemia with the reversible inhibitor amobarbital protects rat heart mitochondria. J Pharmacol Exp Ther 316: 200–207 doi:10.1124/jpet.105.091702. 16174799 10.1124/jpet.105.091702

[5] Burwell LS, Nadtochiy SM, Brookes PS (2009) Cardioprotection by metabolic shut-down and gradual wake-up. J Mol Cell Cardiol 46: 804–810 doi:10.1016/j.yjmcc.2009.02.026. 19285082 10.1016/j.yjmcc.2009.02.026 PMC2683198

[6] Yellon DM, Hausenloy DJ (2007) Myocardial reperfusion injury. N Engl J Med 357: 1121–1135 doi:10.1056/NEJMra071667. 17855673 10.1056/NEJMra071667

[7] Vogt AM, Poolman M, Ackermann C, Yildiz M, Schoels W, et al (2002) Regulation of glycolytic flux in ischemic preconditioning. A study employing metabolic control analysis. J Biol Chem 277: 24411–24419 doi:10.1074/jbc.M201138200. 12006584 10.1074/jbc.M201138200

[8] Lowell BB, Shulman GI (2005) Mitochondrial dysfunction and type 2 diabetes. Science 307: 384–387 doi:10.1126/science.1104343. 15662004 10.1126/science.1104343

[9] Støttrup NB, Løfgren B, Birkler RD, Nielsen JM, Wang L, et al (2010) Inhibition of the malate-aspartate shuttle by pre-ischaemic aminooxyacetate loading of the heart induces cardioprotection. Cardiovasc Res 88: 257–266 doi:10.1093/cvr/cvq205. 20562422 10.1093/cvr/cvq205

[10] Nielsen TT, Støttrup NB, Løfgren B, Bøtker HE (2011) Metabolic fingerprint of ischaemic cardioprotection: importance of the malate-aspartate shuttle. Cardiovasc Res 91: 382–391 doi:10.1093/cvr/cvr051. 21349875 10.1093/cvr/cvr051

[11] Dalgas C, Povlsen JA, Løfgren B, Erichsen SB, Bøtker HE (2012) Effects of fatty acids on cardioprotection by pre-ischaemic inhibition of the malate-aspartate shuttle. Clin Exp Pharmacol Physiol 39: 878–885 doi:10.1111/j.1440-1681.2012.05749.x. 22831462 10.1111/j.1440-1681.2012.05749.x

[12] Contreras L, Gomez-Puertas P, Iijima M, Kobayashi K, Saheki T, et al (2007) Ca2+ Activation kinetics of the two aspartate-glutamate mitochondrial carriers, aralar and citrin: role in the heart malate-aspartate NADH shuttle. J Biol Chem 282: 7098–7106 doi:10.1074/jbc.M610491200. 17213189 10.1074/jbc.M610491200

[13] Palmieri L, Pardo B, Lasorsa FM, del Arco A, Kobayashi K, et al (2001) Citrin and aralar1 are Ca(2+)-stimulated aspartate/glutamate transporters in mitochondria. EMBO J 20: 5060–5069 doi:10.1093/emboj/20.18.5060. 11566871 10.1093/emboj/20.18.5060 PMC125626

[14] Rubi B, Del Arco A, Bartley C, Satrústegui J, Maechler P (2004) The malate-aspartate NADH shuttle member Aralar1 determines glucose metabolic fate, mitochondrial activity, and insulin secretion in beta cells. J Biol Chem 279: 55659–55666 doi:10.1074/jbc.M409303200. 15494407 10.1074/jbc.M409303200

[15] Ralphe JC, Bedell K, Segar JL, Scholz TD (2005) Correlation between myocardial malate/aspartate shuttle activity and EAAT1 protein expression in hyper- and hypothyroidism. Am J Physiol Heart Circ Physiol 288: H2521–H2526 doi:10.1152/ajpheart.00991.2004. 15615843 10.1152/ajpheart.00991.2004

[16] Povlsen JA, Løfgren B, Rasmussen LE, Nielsen JM, Nørregaard R, et al (2009) Cardioprotective effect of L-glutamate in obese type 2 diabetic Zucker fatty rats. Clin Exp Pharmacol Physiol 36: 892–898 doi:10.1111/j.1440-1681.2009.05166.x. 19298538 10.1111/j.1440-1681.2009.05166.x

[17] Løfgren B, Povlsen JA, Rasmussen LE, Støttrup NB, Solskov L, et al (2010) Amino acid transamination is crucial for ischaemic cardioprotection in normal and preconditioned isolated rat hearts–focus on L-glutamate. Exp Physiol 95: 140–152 doi:10.1113/expphysiol.2009.049452. 19717487 10.1113/expphysiol.2009.049452

[18] Birkler RID, Støttrup NB, Hermannson S, Nielsen TT, Gregersen N, et al (2010) A UPLC-MS/MS application for profiling of intermediary energy metabolites in microdialysis samples–a method for high-throughput. J Pharm Biomed Anal 53: 983–990 doi:10.1016/j.jpba.2010.06.005. 20634014 10.1016/j.jpba.2010.06.005

[19] Bolukoglu H, Goodwin GW, Guthrie PH, Carmical SG, Chen TM, et al (1996) Metabolic fate of glucose in reversible low-flow ischemia of the isolated working rat heart. Am J Physiol 270: H817–H826. 8780175 10.1152/ajpheart.1996.270.3.H817

[20] Katz J, Dunn A (1967) Glucose-2-t as a tracer for glucose metabolism. Biochemistry 6: 1–5. 6030319 10.1021/bi00853a001

[21] Doenst T, Taegtmeyer H (2000) Kinetic differences and similarities among 3 tracers of myocardial glucose uptake. J Nucl Med 41: 488–492. 10716324

[22] Neely JR, Denton RM, England PJ, Randle PJ (1972) The effects of increased heart work on the tricarboxylate cycle and its interactions with glycolysis in the perfused rat heart. Biochem J 128: 147–159. 5085551 10.1042/bj1280147 PMC1173579

[23] Bøtker HE, Randsbaek F, Hansen SB, Thomassen A, Nielsen TT (1995) Superiority of acid extractable glycogen for detection of metabolic changes during myocardial ischaemia. J Mol Cell Cardiol 27: 1325–1332. 8531215 10.1016/s0022-2828(05)82395-8

[24] Xu G, Takashi E, Kudo M, Ishiwata T, Naito Z (2004) Contradictory effects of short- and long-term hyperglycemias on ischemic injury of myocardium via intracellular signaling pathway. Exp Mol Pathol 76: 57–65. 14738870 10.1016/j.yexmp.2003.08.003

[25] Wang P, Chatham JC (2004) Onset of diabetes in Zucker diabetic fatty (ZDF) rats leads to improved recovery of function after ischemia in the isolated perfused heart. Am J Physiol Endocrinol Metab 286: E725–E736 doi:10.1152/ajpendo.00295.2003. 14722022 10.1152/ajpendo.00295.2003

[26] Tosaki A, Engelman DT, Engelman RM, Das DK (1996) The evolution of diabetic response to ischemia/reperfusion and preconditioning in isolated working rat hearts. Cardiovasc Res 31: 526–536. 8689644

[27] Ravingerová T, Neckár J, Kolár F (2003) Ischemic tolerance of rat hearts in acute and chronic phases of experimental diabetes. Mol Cell Biochem 249: 167–174. 12956412 10.1023/a:1024751109196

[28] Balakumar P, Sharma NK (2012) Healing the diabetic heart: does myocardial preconditioning work? Cell Signal 24: 53–59 doi:10.1016/j.cellsig.2011.09.007. 21945408 10.1016/j.cellsig.2011.09.007

[29] Baynes J, Murray DB (2009) Cardiac and renal function are progressively impaired with aging in Zucker diabetic fatty type II diabetic rats. Oxid Med Cell Longev 2: 328–334 doi:10.4161/oxim.2.5.9831. 20716921 10.4161/oxim.2.5.9831 PMC2835922

[30] Fredersdorf S, Thumann C, Ulucan C, Griese DP, Luchner A, et al (2004) Myocardial hypertrophy and enhanced left ventricular contractility in Zucker diabetic fatty rats. Cardiovasc Pathol 13: 11–19 doi:10.1016/S1054-8807(03)00109-1. 14761780 10.1016/S1054-8807(03)00109-1

[31] Kupai K, Csonka C, Fekete V, Odendaal L, van Rooyen J, et al (2009) Cholesterol diet-induced hyperlipidemia impairs the cardioprotective effect of postconditioning: role of peroxynitrite. Am J Physiol Heart Circ Physiol 297: H1729–H1735 doi:10.1152/ajpheart.00484.2009. 19734363 10.1152/ajpheart.00484.2009

[32] Ungi I, Ungi T, Ruzsa Z, Nagy E, Zimmermann Z, et al (2005) Hypercholesterolemia attenuates the anti-ischemic effect of preconditioning during coronary angioplasty. Chest 128: 1623–1628 doi:10.1378/chest.128.3.1623. 16162767 10.1378/chest.128.3.1623

[33] Speechly-Dick ME, Baxter GF, Yellon DM (1994) Ischaemic preconditioning protects hypertrophied myocardium. Cardiovasc Res 28: 1025–1029. 7954588 10.1093/cvr/28.7.1025

[34] Ebrahim Z, Yellon DM, Baxter GF (2007) Attenuated cardioprotective response to bradykinin, but not classical ischaemic preconditioning, in DOCA-salt hypertensive left ventricular hypertrophy. Pharmacol Res 55: 42–48 doi:10.1016/j.phrs.2006.10.004. 17079163 10.1016/j.phrs.2006.10.004

[35] Byrne CJ, McCafferty K, Kieswich J, Harwood S, Andrikopoulos P, et al (2012) Ischemic conditioning protects the uremic heart in a rodent model of myocardial infarction. Circulation 125: 1256–1265 doi:10.1161/CIRCULATIONAHA.111.055392. 22319109 10.1161/CIRCULATIONAHA.111.055392

[36] Kocsis GF, Sárközy M, Bencsik P, Pipicz M, Varga ZV, et al (2012) Preconditioning protects the heart in a prolonged uremic condition. Am J Physiol Heart Circ Physiol 303: H1229–H1236 doi:10.1152/ajpheart.00379.2012. 22982778 10.1152/ajpheart.00379.2012

[37] Chatham JC, Marchase RB (2010) The role of protein O-linked beta-N-acetylglucosamine in mediating cardiac stress responses. Biochim Biophys Acta 1800: 57–66 doi:10.1016/j.bbagen.2009.07.004. 19607882 10.1016/j.bbagen.2009.07.004 PMC2814923

[38] Chen H, Shen W-L, Wang X-H, Chen H-Z, Gu J-Z, et al (2006) Paradoxically enhanced heart tolerance to ischaemia in type 1 diabetes and role of increased osmolarity. Clin Exp Pharmacol Physiol 33: 910–916 doi:10.1111/j.1440–1681.2006.04463.x. 17002667 10.1111/j.1440-1681.2006.04463.x

[39] Naito Z, Takashi E, Xu G, Ishiwata T, Teduka K, et al (2003) Different influences of hyperglycemic duration on phosphorylated extracellular signal-regulated kinase 1/2 in rat heart. Exp Mol Pathol 74: 23–32. 12645629 10.1016/s0014-4800(03)80005-9

[40] Oliveira PJ (2005) Cardiac mitochondrial alterations observed in hyperglycaemic rats–what can we learn from cell biology? Curr Diabetes Rev 1: 11–21. 18220578 10.2174/1573399052952578

[41] Finegan BA, Lopaschuk GD, Gandhi M, Clanachan AS (1995) Ischemic preconditioning inhibits glycolysis and proton production in isolated working rat hearts. Am J Physiol 269: H1767–H1775. 7503276 10.1152/ajpheart.1995.269.5.H1767

[42] Halestrap AP, Clarke SJ, Khaliulin I (2007) The role of mitochondria in protection of the heart by preconditioning. Biochim Biophys Acta 1767: 1007–1031 doi:10.1016/j.bbabio.2007.05.008. 17631856 10.1016/j.bbabio.2007.05.008 PMC2212780

[43] Petersen KF, Befroy D, Dufour S, Dziura J, Ariyan C, et al (2003) Mitochondrial dysfunction in the elderly: possible role in insulin resistance. Science 300: 1140–1142 doi:10.1126/science.1082889. 12750520 10.1126/science.1082889 PMC3004429

[44] Pisarenko O, Studneva I, Khlopkov V, Solomatina E, Ruuge E (1988) An assessment of anaerobic metabolism during ischemia and reperfusion in isolated guinea pig heart. Biochim Biophys Acta 934: 55–63. 3378059 10.1016/0005-2728(88)90119-3

[45] Schroeder MA, Atherton HJ, Ball DR, Cole MA, Heather LC, et al (2009) Real-time assessment of Krebs cycle metabolism using hyperpolarized 13C magnetic resonance spectroscopy. FASEB J 23: 2529–2538 doi:10.1096/fj.09–129171. 19329759 10.1096/fj.09-129171 PMC2717776

[46] Taegtmeyer H (1978) Metabolic responses to cardiac hypoxia. Increased production of succinate by rabbit papillary muscles. Circ Res 43: 808–815. 709743 10.1161/01.res.43.5.808

[47] Ashrafian H, Czibik G, Bellahcene M, Aksentijević D, Smith AC, et al (2012) Fumarate is cardioprotective via activation of the Nrf2 antioxidant pathway. Cell Metab 15: 361–371 doi:10.1016/j.cmet.2012.01.017. 22405071 10.1016/j.cmet.2012.01.017 PMC3314920

[48] Randle PJ, England PJ, Denton RM (1970) Control of the tricarboxylate cycle and its interactions with glycolysis during acetate utilization in rat heart. Biochem J 117: 677–695. 5449122 10.1042/bj1170677 PMC1179018

[49] Lesnefsky EJ, Chen Q, Moghaddas S, Hassan MO, Tandler B, et al (2004) Blockade of electron transport during ischemia protects cardiac mitochondria. J Biol Chem 279: 47961–47967 doi:10.1074/jbc.M409720200. 15347666 10.1074/jbc.M409720200

[50] Gaster M (2009) Reduced TCA flux in diabetic myotubes: A governing influence on the diabetic phenotype? Biochem Biophys Res Commun 387: 651–655 doi:10.1016/j.bbrc.2009.07.064. 19615969 10.1016/j.bbrc.2009.07.064

[51] Bradamante S, Marchesani A, Barenghi L, Paracchini L, de Jonge R, et al (2000) Glycogen turnover and anaplerosis in preconditioned rat hearts. Biochim Biophys Acta 1502: 363–379. 11068179 10.1016/s0925-4439(00)00060-0

[52] MacDonald MJ, Longacre MJ, Langberg E-C, Tibell A, Kendrick MA, et al (2009) Decreased levels of metabolic enzymes in pancreatic islets of patients with type 2 diabetes. Diabetologia 52: 1087–1091 doi:10.1007/s00125-009-1319-6. 19296078 10.1007/s00125-009-1319-6 PMC2903059

[53] Ussher JR, Wang W, Gandhi M, Keung W, Samokhvalov V, et al (2012) Stimulation of glucose oxidation protects against acute myocardial infarction and reperfusion injury. Cardiovasc Res 94: 359–369 doi:10.1093/cvr/cvs129. 22436846 10.1093/cvr/cvs129

[54] Li N, Ragheb K, Lawler G, Sturgis J, Rajwa B, et al (2003) Mitochondrial complex I inhibitor rotenone induces apoptosis through enhancing mitochondrial reactive oxygen species production. J Biol Chem 278: 8516–8525 doi:10.1074/jbc.M210432200. 12496265 10.1074/jbc.M210432200

[55] Sárközy M, Zvara A, Gyémánt N, Fekete V, Kocsis GF, et al (2013) Metabolic syndrome influences cardiac gene expression pattern at the transcript level in male ZDF rats. Cardiovasc Diabetol 12: 16 doi:10.1186/1475-2840-12-16. 23320804 10.1186/1475-2840-12-16 PMC3599923

[56] Lopaschuk GD, Wambolt RB, Barr RL (1993) An imbalance between glycolysis and glucose oxidation is a possible explanation for the detrimental effects of high levels of fatty acids during aerobic reperfusion of ischemic hearts. J Pharmacol Exp Ther 264: 135–144. 8380856

[57] Hausenloy DJ, Yellon DM (2007) Reperfusion injury salvage kinase signalling: taking a RISK for cardioprotection. Heart Fail Rev 12: 217–234 doi:10.1007/s10741-007-9026-1. 17541822 10.1007/s10741-007-9026-1

[58] Leonard BL, Watson RN, Loomes KM, Phillips ARJ, Cooper GJ (2005) Insulin resistance in the Zucker diabetic fatty rat: a metabolic characterisation of obese and lean phenotypes. Acta Diabetol 42: 162–170 doi:10.1007/s00592-005-0197-8. 16382303 10.1007/s00592-005-0197-8

[59] Yakubu-Madus FE, Johnson WT, Zimmerman KM, Dananberg J, Steinberg MI (1999) Metabolic and hemodynamic effects of moxonidine in the Zucker diabetic fatty rat model of type 2 diabetes. Diabetes 48: 1093–1100. 10331415 10.2337/diabetes.48.5.1093

[60] Bonen A, Holloway GP, Tandon NN, Han X-X, McFarlan J, et al (2009) Cardiac and skeletal muscle fatty acid transport and transporters and triacylglycerol and fatty acid oxidation in lean and Zucker diabetic fatty rats. Am J Physiol Regul Integr Comp Physiol 297: R1202–R1212 doi:10.1152/ajpregu.90820.2008. 19675275 10.1152/ajpregu.90820.2008

